# Navigating the shift towards sustainable digital building permits and building logbooks

**DOI:** 10.12688/openreseurope.18553.1

**Published:** 2025-03-31

**Authors:** Rita Lavikka, Judith Fauth, Mayte Toscano, Gonçal Costa, Thomas Beach, Pedro Meda Magalhães, Jantien Stoter, Stefanie Brigitte Deac Kaiser, Jeroen Werbrouck

**Affiliations:** 1 Built Environment and Mobility, VTT Technical Research Centre of Finland Ltd, Espoo, Uusimaa, 1000, Finland; 2 University of Cambridge, Cambridge, JJ Thomson Avenue 7, CB3 0RB, UK; 3 Open Geospatial Consortium EU, Seville, Spain; 4 Human Environment Research (HER), La Salle, Ramon Llull University, Barcelona, Spain; 5 School of Engineering, Cardiff University, Cardiff, UK; 6 CONSTRUCT/Gequaltec, Faculty of Engineering, University of Porto, Porto, Portugal; 7 Delft University of Technology, Delft, The Netherlands; 8 Politehnica University of Timisoara, Timișoara, Timiș County, Romania; 9 Ghent University, Ghent, Flanders, Belgium

**Keywords:** Digital building logbook (DBL), digital building permit (DBP), sustainable construction, sustainable building management, sustainable development goals, SDG, data-driven

## Abstract

The architecture, engineering, construction, and operation sectors face significant sustainability challenges. Environmentally, it contributes significantly to greenhouse gas emissions and resource depletion. Socially, it must address issues such as worker safety and community impact. Economically, the sector struggles to balance cost efficiency with sustainable practices. Digital solutions are expected to support sustainable construction. Digital building permits (DBP) and digital building logbooks (DBL) provide examples of digital solutions that support sustainable construction and building management. DBP and DBL are intertwined to enhance the efficiency and transparency of the construction and building management processes. However, research on how DBP and DBL can address sustainability in practice is limited. To address this research gap, this study uses the UN’s Sustainable Development Goals (SDGs) as an analytical framework for the sustainability of DBP and DBL. The research consisted of four phases. First, an expert group identified and selected the SDGs related to DBP and DBL. Then, the experts identified the relevant targets of the selected SDGs. Subsequently, the expert group specified the DBP and DBL practices that supported the relevant targets. Finally, the expert group organised a workshop with external experts in the study area to verify the practices supporting the SDGs. The study identified DBP and DBL practices contributing to achieving 10 SDGs: 3, 7, 8, 9, 10, 11, 12, 13, 16, and 17. The findings suggest that DBL and DBL practices provide opportunities for environmental, social, and economic sustainability; however, further empirical research is needed. The study concluded that DBP and DBL practices can enhance energy management, reduce carbon emissions, improve resource utilisation, and reduce waste. They also support creating a built environment that is user-friendly and remotely accessible, as well as offering financial benefits and improving efficiency and transparency while minimising errors from human interpretation through automation.

## Plain language summary

The Architecture, Engineering, Construction, and Operations sector faces several big challenges when it comes to sustainability. Environmentally, it is a major contributor to greenhouse gas emissions and uses up a lot of natural resources. Socially, it needs to ensure worker safety and consider the impact on local communities. Economically, the sector often struggles to find a balance between keeping costs low and adopting sustainable practices.

Digital solutions are seen as a way to help make construction more sustainable. Two examples of these solutions are digital building permits (DBP) and digital building logbooks (DBL). These tools are designed to make the construction process more efficient and transparent. However, there isn’t much research on how well these tools actually promote sustainability.

To fill this gap, a study was conducted using the United Nations’ Sustainable Development Goals (SDGs) as an analysis framework. The research was done in four phases. First, experts identified which SDGs were relevant to DBP and DBL. Then, they pinpointed specific targets within those Goals. After that, the experts specified DBP and DBL practices supporting the relevant targets. Finally, a workshop was held to confirm how DBP and DBL practices support these goals.

The study found that DBP and DBL practices contribute to achieving several SDGs, including good health and well-being, affordable and clean energy, decent work and economic growth, industry innovation and infrastructure, sustainable cities and communities, responsible consumption and production, climate action, peace, justice, and strong institutions, and partnerships for the goals. The study concludes that DBP and DBL practices can enhance energy management, reduce carbon emissions, improve resource utilisation and reduce waste. They also support creating a built environment that is user-friendly and remotely accessible, as well as offering financial benefits and improving efficiency and transparency while minimising errors from human interpretation through automation.

## Introduction

The architecture, engineering, construction, and operation (AECO) sector faces several sustainability challenges. Environmentally, it contributes significantly to greenhouse gas emissions and the depletion of natural resources ( Kanafani *et al.*, 2023). Socially, the AECO sector faces issues such as ensuring worker safety and minimizing negative impacts on local communities ( Zhang *et al.*, 2020). Economically, the sector often struggles to balance cost efficiency with adopting sustainable practices ( Alaloul *et al.*, 2022; Hussain & Hussain, 2023). Therefore, sustainable construction and building management are necessary to address these challenges.

Digital transformation has been shown to support sustainability transformation ( Chatzistamoulou, 2023). In the AECO sector, digital building permit (DBP) processes and digital building logbooks (DBL) can contribute to supporting sustainable construction and building management ( Crisan *et al.*, 2024). The DBP process involves the use of digital tools and online building permitting and compliance services to streamline and automate the preparation, review, and approval of building permits ( Ataide *et al.*, 2023; Fauth *et al.*, 2024; Ullah *et al.*, 2022). The DBP process was found to be more efficient, faster, and transparent ( Noardo *et al.*, 2022). On the other hand, DBL encompasses all pertinent building-related data throughout the building’s entire lifecycle, offering various stakeholders the specific information they need for different purposes at the appropriate times ( Malinovec Puček *et al.*, 2023).

DBP and DBL concepts are intertwined throughout the building lifecycle ( Mêda *et al.*, 2024a). For example, when a DBP process begins, DBL data can be used to provide the required datasets. On the other hand, when a DBP is issued, the information can potentially be automatically recorded in the DBL, ensuring that all relevant data about the construction project, including compliance with regulations, are documented from the start. Data-driven is a commonality between the two concepts; the foundational element intersects with several other components, and the relation to BIM is found to be crucial. From an incremental perspective, DBL plays a key role as a Digital Twin enabler at the building scale ( Mêda *et al.*, 2021). As the building enters the in-use phase, several services and purposes are expected to be provided by the DBL, whereas real-time data are captured and versioned if required, reflecting any changes or inspections related to the permit ( European Commission, 2021; Mêda *et al.*, 2024a; Mêda *et al.*, 2024b; Volt & Toth, 2020).

The availability of consistent and reliable building data can contribute to better design, construction, and management of buildings. Currently, data regarding the physical characteristics of buildings, including information on environmental performance, sustainability, and the data necessary for checking building code compliance, remain unreliable, scarce, and not very accessible. Together, DBP and DBL can establish a common approach that aggregates all related data on a building, such as building materials and energy usage, helping to identify inefficiencies and to take corrective measures to reduce waste ( Mêda *et al.*, 2024a). Building-related data can also help better manage building maintenance by identifying the potential risks related to the lifespan of building systems and materials ( European Commission, 2021; Mêda *et al.*, 2024a; Mêda *et al.*, 2024b; Volt & Toth, 2020). In addition, non-digital or paper-based systems require extensive paperwork, multiple physical copies, consumption of other resources for their generation, and numerous in-person visits to various government offices. By contrast, a digital system eliminates the need for physical documents, reduces paper usage, and conserves the trees and water used in paper production. Moreover, by streamlining administrative tasks, digital systems can reduce the energy consumption associated with running a physical office.

Thus, DBP and DBL have the potential to enhance the efficiency, transparency, and sustainability of the construction and building management processes. They promise more precise planning and efficient resource use, reduced waste, and improved building energy management ( Crisan *et al.*, 2024; Mêda *et al.*, 2024a; Mêda *et al.*, 2024b; Noardo *et al.*, 2022). However, evidence-based research on the sustainability of DBP and DBL is limited. Several studies have mentioned that DBP processes enhance sustainability ( Amor & Dimyadi, 2021; Malinovec Puček *et al.*, 2023), but they do not explicitly study how and to what extent. Instead, they considered it to be an implicit consequence of the DBP process and DBL implementation. This presents a key research gap in that, thus far, no study has empirically examined the sustainability impacts of DBP and DBL.

To address this research gap, the present study aims to answer the following research question: *What are the sustainability impacts of DBL and DBP and how can those impacts be achieved?* It does this by taking the UN’s Sustainable Development Goals (SDGs) ( United Nations, 2024) as an analytical framework to study the sustainability of DBP and DBL. Therefore, this study contributes to sustainable construction and building management by identifying DBP and DBL practices that contribute to sustainability. This study sets the stage for further research on sustainability in the context of DBP and DBL.

## Research process

**Figure 1.**
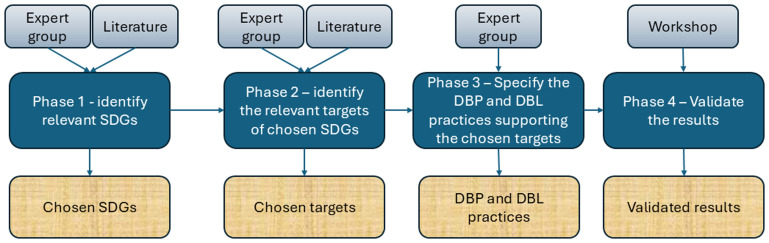
Research process: data collection methods, research phases and outputs.

The research consisted of four phases ( Figure 1). In the first phase, the authors, considered as an expert group, searched for literature on the relationship between sustainability and DBP and DBL. The SDGs work as a sustainability analysis framework for structured identification. SDGs consist of 17 goals, each with specific targets to be achieved by 2030. In total, there were 169 targets across all SDGs. During this first phase, the expert group analyzed the relationship between DBP processes and SDGs and between DBL and SDGs. Following an autoethnographic approach ( Grosse, 2019), the expert group used their experience and knowledge to identify which SDGs were related to DBP and DBL. The expert group consists of researchers who have studied DBP processes, DBLs, or both for at least two years, and most are involved in ongoing projects and collaborations on the topics.

The first round of analysis led to some dissenting opinions among the expert group, but after discussions, ten SDGs were selected to be related to DBP and/or DBL: 3, 7, 8, 9, 10, 11, 12, 13, 16, and 17.

The second phase focused on identifying relevant targets of the selected SDGs. The selected Goals 3, 7, 8, 9, 11, 12, 13, 16 and 17 have 95 Targets. The expert group also searched for evidence-based sources – reports, scientific articles, and regulatory documents – to support their claims on the relationships. This phase provides a list of selected targets for the selected SDGs.

In the third phase, the expert group specified DBP and DBL practices to support the relevant targets, underlined with references where possible. The references ranged from scientific literature and project reports to practical and project experience.

In Phase 4, the expert group arranged a workshop to validate the results. The hybrid workshop had 38 participants, approximately half of whom participated onsite and the rest online. The workshop was conducted on September 24, 2024, at the Sustainable Places 2024 conference in Luxembourg. Participants were given a research information sheet explaining the research and their rights. Personal data were not collected, but some background information on the participants was collected anonymously using Slido ^1^ to ensure that the participants were competent in validating the study results. For example, participants were asked about their country of origin, professional title, and level of knowledge of DBP and DBL. Most of the participants worked in R&D related to the built environment. The professional titles included project managers, research associates, researchers, R&D directors, and a professor. On a scale of 1 (no knowledge), 2 (some knowledge), 3 (lots of knowledge), and 4 (experts in the field), participants’ levels of knowledge of DBP and DBL varied. However, most participants had significant knowledge of either DBPs or DBLs. The participants came from the following European countries and regions: Luxembourg, Portugal, the UK, the Netherlands, Spain (including Catalonia), France, Italy, Poland, Germany, and Belgium.

The workshop consisted of two parts. The first part, 1.5 hours, included an introduction consisting of the research information sheet (10 min), a presentation of SDGs (20 min), and six project presentations on DBP and DBL from ongoing R&D projects (60 min). After the first part, there was a 30-minute coffee break. The second part of the workshop started with collecting the background information on the participants with Slido (20 min) and then continued with group discussions to validate the connection between SDGs and DBP processes and DBLs (70 min). The participants were not shown the analysis that the expert group had done, but they had the opportunity to make their observations, which the expert group then compared with its own findings after the workshop. During the group discussions, the participants could select from three options to indicate how they saw the relationship between each SDG and DBP and each SDG and DBL: 0 (no relationship), 1 (implicitly related), or 2 (explicitly related). The exercise was performed on Miro ^2^ which included two tables: one for analyzing the relationship between SDGs and DBP and one table for analyzing the relationship between SDGs and DBL. Both tables’ columns included the scale numbers (0, 1, and 2) and rows included the 17 SDGs. Mainly the discussion facilitators made notes to tables based on the group discussions. However, the participants could also access the tables themselves, but only a few added any content there. 

## Results

This section reports findings regarding the relationship between SDGs and DBP and/or DBL. SDGs 7, 9, 11, and 13 had the most evident relationship to DBP and DBL practices. The results originated from the expert group and workshop discussions. The workshop participants agreed that DBP processes and DBLs can enhance sustainability in the AECO sector. They pointed out how DBP and DBL can improve the traceability of materials, reduce environmental impacts, and promote circularity in construction. The participants strongly emphasized data transparency and interoperability, which could further streamline sustainable practices. These workshop findings were aligned with those of the expert group.

Supplementary Table 1 lists those SDGs and targets (target descriptions are directly quoted) that were identified as linked to sustainable DBP or DBL practices through evidence-based references (marked after the name of the goal), the expert group and/or workshop discussions. The right column of Table 1 describes the DBP and DBL practices that contribute to achieving ten SDGs: 3 (Good Health and Well-Being), 7 (Affordable and Clean Energy), 8 (Decent Work and Economic Growth), 9 (Industry, Innovation, and Infrastructure), 10 (Reduced Inequalities), 11 (Sustainable Cities and Communities), 12 (Responsible Consumption and Production), 13 (Climate Action), 16 (Peace, Justice, and Strong Institutions) and 17 (Partnerships for the Goals). 

The following paragraphs briefly summarise the findings regarding the relationship between DBP and DBL to the targets of the selected SDGs and sustainable practices.

Regarding SDG 3, “Good Health and Well-Being,” and its chosen targets of 3.4, 3.6, 3.8, and 3.9, it was found that DBP processes can support universal health coverage by ensuring that health facilities are safe and comply with health standards ( Soliman-Junior *et al.*, 2020; Soliman-Junior *et al.*, 2021). DBL, a Digital Twin enabler, captures data related to soil properties, toxicity of used materials, and building air quality, thereby supporting users’ health and safety. In general, digital services have ensured access to public services despite physical restrictions during the COVID-19 pandemic, highlighting the importance of digital systems in crises.

Regarding SDG 7, “Affordable and Clean Energy,” and its chosen Targets 7.1-7.3, it was found that DBPs, while not directly related to energy services, influence the planning and construction of energy-efficient buildings, and the incorporation of renewable energy sources such as geo-energy ( Majuri *et al.*, 2020). Building codes and regulations within the permit process can mandate energy-efficient designs, materials, and technologies, thereby reducing energy consumption. However, research indicates discrepancies between the permitted energy consumption calculations and actual measurements ( Ruusala *et al.*, 2018). Permits also facilitate the installation of renewable energy systems, thereby contributing to a sustainable energy mix ( Chen *et al.*, 2024; Li & Feng, 2023). Regarding the SDG7 targets, 7.a and 7.b, DBP processes are national mechanisms with some similarities ( Bloch & Fauth, 2023; Fauth *et al.*, 2023a; Fauth *et al.*, 2023b; Fauth & Soibelman, 2022; Noardo *et al.*, 2022). They can facilitate knowledge transfer and capacity building, which are essential for deploying energy efficiency and renewable energy technologies. Enforcing regulations for energy-efficient materials and technologies through building permits promotes clean energy adoption and supports investment in energy infrastructure.

Regarding SDG 8, “Decent Work and Economic Growth,” and its chosen Targets 8.1-8.5, it was identified that DBP processes ensure safety and regulatory compliance while also boosting economic productivity by fostering innovation, efficiency, and the use of sustainable materials ( Fauth *et al.*, 2024). Digital technologies can streamline the permitting process, reduce bureaucratic barriers, and facilitate SME growth ( Ataide *et al.*, 2023; Beach *et al.*, 2024; Beach *et al.*, 2020). This can boost economic activity and job creation ( World Bank Group, 2020; World Bank Group, 2024). Digitalisation enhances transparency and efficiency, making regulatory navigation easier for entrepreneurs and simplifying compliance. Digitalising the building permit process reduces paper usage and streamlines operations, leading to efficient resource use. It enhances data collection and analysis, and informs sustainable urban planning and construction. Digitalisation also improves monitoring and enforcement of environmental regulations, creates tech job opportunities, and improves service access for more people.

Regarding SDG 9, “Industry, Innovation, and Infrastructure,” and its chosen targets 9.1-9.5, it was discovered that DBP supports resilient infrastructure by enhancing project efficiency and transparency and reducing delays and costs. This ensures that new constructions meet modern sustainability and resilience standards, foster innovation through smart technologies, and ensure compliance with building codes and safety regulations. Automated code compliance checks verify adherence to seismic and sustainability standards ( Amor & Dimyadi, 2021; Patlakas *et al.*, 2024). DBP processes support digital transformation, improve resource-use efficiency, and encourage clean and environmentally sound technologies and industries ( Ataide *et al.*, 2023; Crisan *et al.*, 2024). Innovations such as applied computing for code compliance checking can boost the R&D workforce and increase public and private R&D spending ( Amor & Dimyadi, 2021).

Regarding SDG 10, “Reduced Inequalities,” and its chosen Targets 10.2 and 10.3, it was found that DBP processes and DBL provide insights into built stock, its condition, occupancy, and costs, aiding decision making. Thus, they enhance transparency, reduce discriminatory practices, and ensure equal opportunities.

Regarding SDG 11, “Sustainable Cities and Communities,” and its chosen targets 11.1-11.7, and 11. a, the analysis showed that DBP processes streamlined housing project approvals, facilitating affordable housing development and access to basic services, leading to efficient resource use and faster housing delivery. Digitalising the permit process enhances urban planning efficiency and transparency, facilitates citizen participation and developer compliance ( Eirinaki *et al.*, 2018). Digital processes enhance transparency and accountability, ensuring that buildings meet sustainability and resilience standards. Streamlining the permit process reduces time and cost, aiding developers in the least-developed countries. This fosters efficient use of local materials and sustainable building practices ( cdbb, 2019; Olanrewaju Yusuf *et al.*, 2021).

Regarding SDG 12, “Responsible Consumption and Production”, and its chosen Targets 12.2 and 12.5-12.8, it was discovered that a digital permit process reduces the environmental impact of construction by minimising waste, optimising material use, and ensuring efficient resource use. It also provides better data for resource management and mandates the reuse of materials. Enhanced accessibility and transparency promote awareness of sustainable practices and regulations ( Hradil *et al.*, 2024), aligning with development goals. Selective demolition permits the reuse of recoverable materials based on data availability and DBL.

Regarding SDG 13, “Climate Action,” and its chosen Targets 13.1-13.3, digitalising the permit process can reduce construction’s environmental impact by minimising waste, optimising material use, and ensuring efficient resource use. It also provides improved data and analytics for resource management ( Gillingham *et al.*, 2021; Hradil, 2023; Hradil *et al.*, 2024).

Regarding SDG 16¸ “Peace, Justice, and Strong Institutions,” and its chosen targets of 16.6, 16.7, and 16.10, it was identified that digitalising the permit process improves transparency in government spending and budget implementation, ensuring effective use of resources for sustainable development ( World Bank Group, 2020; World Bank Group, 2024). It enhances citizen engagement and inclusivity in local governance and promotes transparency and accountability. Digital platforms provide better access to information and facilitate informed public participation in decision-making ( Eirinaki *et al.*, 2018).

Regarding SDG 17, “Partnerships for the Goals” and its chosen Targets 17.1 and 17.6, it was concluded that DBPs and DBLs enhance global collaboration in the building sector by offering a unified platform for data sharing throughout the building lifecycle. This standardisation promotes interoperability and cooperation, thereby aligning sustainability and efficiency efforts worldwide. ( World Bank Group, 2020; World Bank Group, 2024)

## Discussion

In general, digital technologies can support the achievement of the SDGs ( Mantovani Ribeiro *et al.*, 2021). They can help identify and agree on the most sustainable ways to work, build appropriate skills across stakeholder groups, attract finance, and ensure practical processes for multi-stakeholder engagement at all stages of building construction. This study supports these findings in the context of DBP and DBL technologies, which provide opportunities for environmental, social, and economic sustainability. Environmentally, they can help enhance energy management, reduce carbon emissions, and improve resource utilisation and waste reduction. Socially, they can promote social inclusion by creating an accessible, user-friendly, and remotely accessible built environment. Economically, they can offer cost savings and long-term financial benefits, streamlining permit application and building management processes, and improving efficiency and transparency while minimising human error or differences due to human interpretation through automated data handling. However, it needs to be noted that digitalisation also has negative environmental impacts. For example, data storage has environmental impacts due to cooling servers' high energy and water usage. Although solutions such as using renewable energy to power data centres are being studied to counteract this impact, more research is needed to understand the environmental impact of digitalisation.

Although this paper has envisioned a future in which digital means support the achievement of sustainable construction, challenges exist in the implementation of DBP processes and DBLs. These challenges can be grouped into initial implementation costs, data security, user adoption, and interoperability and provide the corresponding legislative framework for its correct deployment. The initial setup costs, which can be significant, include software development, hardware installation, and staff training. Data security and privacy concerns may arise owing to the storage of sensitive building data in a digital format. The digitalisation of building permits varies across Europe. Some regions adopt Artificial Intelligence (AI), whereas others use PDFs. The need for harmonisation at the European level is crucial for efficiency. Furthermore, ensuring compatibility and seamless data exchange between software platforms and building systems presents interoperability challenges. Therefore, solutions are needed to create strategies that support stakeholders in adopting digital solutions, ensuring the protection of sensitive information, addressing potential user resistance, and enhancing digital literacy. Thus, solutions for seamless data integration across platforms are needed, especially when considering multi-asset DBLs (e.g., for infrastructure), consisting of multiple lower-level DBLs. A lack of familiarity with digital tools among building controls, permit applicants and management staff can also hinder user adoption. The digital divide, which affects people living in poverty (SDG 1), especially in rural areas and developing countries, is also a challenge. Thus, an improved digital infrastructure is crucial to ensure equitable access to DBPs and DBLs.

As data-driven concepts, DBP and DBL can reshape the way production, consumption, and living occur. However, data infrastructure platforms and governance frameworks are required to facilitate data pooling, access, and sharing. In the European Union, data spaces will play this role. Following Europe’s strategy for the digital age, the legal framework is being adjusted, and concerning the built environment, the common European Green Deal Data Space ^3^ is developing a highly accurate digital model of Earth, on top of which all other layers will stand. Currently, there are no common data spaces for construction or built environments. This situation may not be an issue if the required data are captured, stored, and managed in several data spaces. Nevertheless, understanding these boundaries may be challenging and raises several issues. However, if a construction/built environment is set in a similar situation with boundaries, defining the borders of this data space might be extremely difficult. All these ongoing discussions are relevant and constitute challenges for the system architecture and data management systems of DBP and DBL. The Rolling Plan for ICT Standardization 2024 ^4^ considers four key processes for all actions: “Data Governance”, “Data Discovery”, “Data Sharing”, and “Data Usage”. All of these need to be evaluated from the data spaces and DBP/DBL perspectives. This paper focused mainly on the “Data Usage” process, namely, supporting the accomplishment of the UN SDGs' Targets, but it is paramount to work with the other processes to tackle the challenges.

## Conclusions

This study addresses the following research question: “ *What are the sustainability impacts of DBL and DBP, and how can those impacts be achieved?*”. To this end, we explored the sustainability of DBP and DBL by applying the UN’s SDGs as an analytical framework.

The findings of this study are that DBP and DBL contribute, both directly and indirectly, to SDGs 3 (Good Health and Well-Being), 7 (Affordable and Clean Energy), 8 (Decent Work and Economic Growth), 9 (Industry, Innovation, and Infrastructure), 11 (Sustainable Cities and Communities), 12 (Responsible Consumption and Production), 13 (Climate Action), 16 (Peace, Justice, and Strong Institutions), and 17 (Partnerships for the Goals). This study documents how these sustainable impacts of DBP and DBL can be achieved. These findings indicate that digitalising building permits and logbooks provides environmental, social, and economic sustainability. However, the effects of DBP and DBL remain to be determined. The value of digitalisation can only be measured once digital transformation has been completed and digitalisation is in place. During the transformation process, the impacts of DBP and DBL are difficult to measure, even though predictions can be made. Overall, the findings indicated that DBP processes and DBL are part of a broader digital transformation that can support sustainability.

The primary impact of this study is that it provides an outline framework and encouragement for DBP and DBL research communities to begin assessing their work against SDGs. This will enable both the quantitative and qualitative assessment of DBP and DBL research as it enters adoption across Europe. However, key future work in this area is needed to further develop and validate methods to assess DBL and DBP implementation against the relevant SDGs.

Additionally, the findings provide implicit evidence that DBP and DBL are increasingly aligned with the three current European initiatives and strategies. One of them is Europe’s fit for the digital age. DBP and DBL support the EU’s digital transformation by standardising data collection, management, and sharing across the construction sector. This harmonisation facilitates transparency, trust, and informed decision-making. Another initiative is the European Green Deal and its Renovation Wave, which aims to improve the energy efficiency of buildings. DBP and DBL can support integrating data from energy performance certificates, smart readiness indicators, and building renovation passports, supporting Green Deal’s goals of reducing carbon emissions and promoting sustainable building practices. Finally, DBL can contribute to the New Circular Economy Action Plan initiative by providing a comprehensive repository of building-related data. These data help track materials and resources, promote reuse and recycling, and support the lifecycle management of buildings. 

## Ethical consideration

Ethical approval and consent were not required.

## Data Availability

Repository name: ACCORD project. https://doi.org/10.5281/zenodo.15078988 This project contains the following extended data: Supplementary Table 1 (The selected targets of the selected SDGs and DBP and DBL practices with evidence-based references) Workshop data (Data collected during a workshop on Digital Building Logbooks and Permit Processes for Sustainability. The workshop was held in Sustainable Places on the 24th of September 2024 in Luxembourg) Data are available under the terms of the Creative Commons Attribution 4.0 International license (CC-BY 4.0) ( https://creativecommons.org/licenses/by/4.0/).
