# Supplementary material for: Navigating the shift towards sustainable digital building permits and building logbooks
Source: Open Res Eur. 2025 Aug 4;5:90. Originally published 2025 Mar 31. [Version 2] doi: 10.12688/openreseurope.18553.2 (PMC12432498; doi:10.12688/openreseurope.18553.2)
Supplement: Peer review history

# Reviewer report: Reviewer response for version 2

**DOI:** 10.21956/openreseurope.22640.r59065

**Published:** 2025-09-12

**Authors:** Jan Holmstrom

**Affiliations:** 1 Aalto University, Espoo, Finland

The revised manuscript is acceptable. Based on a literature review, expert group workshops that paper examines the possible sustainability improvements that digital building permits and logbooks can have. 

 The challenge for researching the topic is that it is an emerging topic, with digitalization ongoing. Because of this the reliance on experts for assessing is appropriate.

Is the study design appropriate and does the work have academic merit?

Yes

Is the work clearly and accurately presented and does it cite the current literature?

Yes

If applicable, is the statistical analysis and its interpretation appropriate?

Not applicable

Are all the source data underlying the results available to ensure full reproducibility?

Yes

Are the conclusions drawn adequately supported by the results?

Yes

Are sufficient details of methods and analysis provided to allow replication by others?

Yes

Reviewer Expertise:

expert in the digitalization of operations

I confirm that I have read this submission and believe that I have an appropriate level of expertise to confirm that it is of an acceptable scientific standard.

# Reviewer report: Reviewer response for version 2

**DOI:** 10.21956/openreseurope.22640.r59062

**Published:** 2025-09-04

**Authors:** Adrian Wildenauer

**Affiliations:** 1 Bern University of Applied Sciences, Bern, Switzerland

Thank you very much indeed for the opportunity to review this interesting paper, which brings up as one of the first papers the connection between digital building permits and building logbooks.

 This research paper by Lavikka et al. addresses an important gap in understanding the sustainability impacts of digital building permits (DBP) and digital building logs (DBL) by systematically mapping these technologies to the United Nations Sustainable Development Goals (SDGs). While the study makes a valuable contribution to this field, some aspects deserve a little more attention.

 Strengths

 The most important contribution of the paper is that it is the first to systematically map DBP and DBL practices to specific SDG goals. The four-phase methodology, which combines expert interviews, SDG mapping, practice documentation, and stakeholder validation through a workshop with 38 participants, is methodologically sound and demonstrates academic rigour.

 The use of the UN SDGs as an analytical framework provides a structured, internationally recognized approach to sustainability assessment, which enhances the credibility of the study and its potential for broader application.

 The research successfully identifies concrete links between digital building technologies and sustainability outcomes, going beyond the existing literature, which treated sustainability as an implicit benefit of digitalization. Validation through a hybrid European workshop lends empirical weight to the expert-based analysis.

 Points for optimization

 Study design and academic value (partially adequate): The study design is fundamentally sound, but limited by its European focus and one-off data collection at a specific point in time. However, it is naturally an important topic in Europe at present. The academic value is evident in the systematic approach and reproducible framework, but the novelty of the results is questionable. The geographical limitation and cross-sectional nature reduce the generalizability of the results. The title is probably poorly chosen here, as the study primarily deals with European issues. This gives it the nimbus of a comprehensive study.

 Methodological details (partially adequate): The section on methodology provides an adequate overview, but important details for complete reproducibility are missing. The most important missing elements include specific procedures for selecting experts, data processing protocols, consensus-building mechanisms, and detailed analysis procedures. The lack of information on data normalization, cleaning methods, and quality control measures significantly impairs reproducibility. Although the tools used (Slido, Miro) are mentioned, the specific analysis procedures are insufficiently documented.

 Data availability (partially accessible): Although the authors provide a Zenodo repository with supplementary materials and workshop data under a Creative Commons licence, there are still gaps. The raw data of the expert opinions, detailed workshop discussions and individual responses of the participants prior to aggregation are not available, which limits the verifiability of the analysis processes. The anonymization process protects privacy, but reduces the transparency of the analytical validation. Nevertheless, an important contribution.

 Support for conclusions (partially adequate): The conclusions follow logically from the study design, but go beyond what the data can fully substantiate. While the identification of relevant SDGs is well validated, there is a lack of empirical evidence for statements on specific implementation mechanisms and quantified benefits. The study documents which practices contribute to sustainability, but does not sufficiently explain how these effects can be achieved in practice. A gap remains between theoretical classification and practical implementation guidance.

 Recommendation

 This work makes a significant contribution to the interface between digital construction technologies and sustainability assessment by providing a structured framework for evaluation. However, the numerous limitations in the key assessment criteria leave considerable room for improvement.

 The work establishes a foundation for future research, but requires improved methodological transparency, expanded geographical scope, and more robust conclusions that acknowledge the preliminary nature of the results while highlighting the valuable contribution of the framework.

Is the study design appropriate and does the work have academic merit?

Partly

Is the work clearly and accurately presented and does it cite the current literature?

Yes

If applicable, is the statistical analysis and its interpretation appropriate?

Not applicable

Are all the source data underlying the results available to ensure full reproducibility?

Partly

Are the conclusions drawn adequately supported by the results?

Partly

Are sufficient details of methods and analysis provided to allow replication by others?

Yes

Reviewer Expertise:

Information Management, Digital Building Permits, Digital Product Passports, Building-as-a-Service

I confirm that I have read this submission and believe that I have an appropriate level of expertise to confirm that it is of an acceptable scientific standard, however I have significant reservations, as outlined above.

# Reviewer report: Reviewer response for version 1

**DOI:** 10.21956/openreseurope.20061.r55473

**Published:** 2025-06-25

**Authors:** Muhammad Shafique

**Affiliations:** 1 Brunel University London, Middlesex, UK

OVERALL REVIEW RESULT

 This is a well-structured and written article. However, as the main drawback, it isn't easy to recognise the importance of the research results. Reading the conclusions, the study has not contributed significantly to new findings that are not already available in the existing literature, and the overall outcome sounds trivial. For example, the authors have ended up stating: "The study documents how these sustainable impacts of DBP and DBL can be achieved"; but how these will be achieved are not explained in details.  Also, I believe it is very important to highlight content on the importance of incorporating methods. 

 I hope that this feedback will be helpful as you look to strengthen your research efforts.

 MORE SPECIFIC COMMENTARY

 The paper aims to answer the following research question: *What are the sustainability impacts of DBL and DBP and how can those impacts be achieved?*

 The aim is to explore sustainable construction and building management by identifying DBP and DBL practices that contribute to sustainability.

 The structure of this paper is suitable.

 The English language used in the manuscript is good enough to convey the idea of the paper without critical mistakes in grammar, word selection, and typing. Still, a second English proofread is suggested.

 Title - It is reasonable.

 Keywords - They are reasonable.

 It is still unclear how the research questions relate to the statements in introduction section and how the current article goes beyond other existing literature in this area. To address this issue, please try to embed the questions into the body of the text presented in introduction section.

 Research Process; 

 It is essential to elaborate on how the experts were selected and which sampling method was used to select experts? 

 It is important to clearly give detail information on methods selection for methodology section. 

 While the work appears technically solid and well-organized, with relevant features and structured categories, the data collection and processing protocols are not described in sufficient technical detail. The article lacks the following:

  ○ Clarification on whether data were normalized or standardized.

 ○ No mention of data cleaning, anonymization, or aggregation methods.

 ○ Uncertainty regarding the temporal resolution of data.

  ○ The tool or platform used for data processing and visual analytics is mentioned vaguely but not named or described.

 Conclusion

 Were there any limitations in your research work?

Is the study design appropriate and does the work have academic merit?

Partly

Is the work clearly and accurately presented and does it cite the current literature?

Yes

If applicable, is the statistical analysis and its interpretation appropriate?

Not applicable

Are all the source data underlying the results available to ensure full reproducibility?

No source data required

Are the conclusions drawn adequately supported by the results?

Yes

Are sufficient details of methods and analysis provided to allow replication by others?

Partly

Reviewer Expertise:

Sustainability, Life-cycle Energy and Environmental Assessment (LCA), Circular Economy, Sustainable Construction

I confirm that I have read this submission and believe that I have an appropriate level of expertise to confirm that it is of an acceptable scientific standard, however I have significant reservations, as outlined above.

# Response

**Published:** 2025-07-08

**Authors:** Rita Lavikka

**Affiliations:** Built Environment and Mobility, VTT Technical Research Centre of Finland Ltd, Espoo, Uusimaa, Finland

We thank the reviewer for the constructive comments, which helped us improve our article. Below, we provide our more detailed responses to each review comment. Review comment: “This is a well-structured and written article. However, as the main drawback, it isn't easy to recognise the importance of the research results. Reading the conclusions, the study has not contributed significantly to new findings that are not already available in the existing literature, and the overall outcome sounds trivial. For example, the authors have ended up stating: "The study documents how these sustainable impacts of DBP and DBL can be achieved"; but how these will be achieved are not explained in details.  Also, I believe it is very important to highlight content on the importance of incorporating methods. I hope that this feedback will be helpful as you look to strengthen your research efforts.” Our response: We thank the reviewer for this thoughtful feedback. In response, we have revised the Discussion and Conclusions sections to more clearly articulate the novelty and significance of our findings. We now emphasise that our study is the first to systematically map Digital Building Permit (DBP) and Digital Building Logbook (DBL) practices to specific UN Sustainable Development Goals (SDGs), providing a structured and replicable framework for sustainability assessment. Please see also Supplementary Table 1. We refined the Conclusions section to answer both research questions explicitly and added a new paragraph on the study's impact: The impact of this study is twofold. First, it introduces a structured and replicable framework for assessing the sustainability of DBP and DBL practices by explicitly mapping them to specific SDG targets. This goes beyond prior literature, which often treats sustainability as an implicit benefit rather than a measurable outcome. Second, the study demonstrates how expert-driven analysis and stakeholder validation can be used to identify actionable DBP and DBL practices that support environmental, social, and economic sustainability. By doing so, the paper lays a foundation for future empirical research and policy development, supporting the alignment of digital construction tools with global sustainability objectives. This will enable both the quantitative and qualitative assessment of DBP and DBL research as it enters adoption across Europe. However, key future work is needed in this area to develop further and validate methods for assessing DBL and DBP implementation against the relevant SDGs. We also added more explanation about the uniqueness of our method to the Research process section. Review comment: “The paper aims to answer the following research question: What are the sustainability impacts of DBL and DBP and how can those impacts be achieved? The aim is to explore sustainable construction and building management by identifying DBP and DBL practices that contribute to sustainability. The structure of this paper is suitable. The English language used in the manuscript is good enough to convey the idea of the paper without critical mistakes in grammar, word selection, and typing. Still, a second English proofread is suggested.” Our response: We corrected grammatical mistakes, shortened long sentences and made a second English proofread. Review comment: “Title - It is reasonable. Keywords - They are reasonable. It is still unclear how the research questions relate to the statements in introduction section and how the current article goes beyond other existing literature in this area. To address this issue, please try to embed the questions into the body of the text presented in introduction section.” Our response: We thank the reviewer for this insightful comment. In response, we have revised the final paragraph of the Introduction to explicitly embed the research question and clarify how it emerges from the identified research gap. We also added a sentence earlier in the Introduction to highlight the novelty of our approach compared to existing literature. These changes aim to improve the logical flow from problem identification to research objective and to better position our contribution within the broader academic discourse. Review comment: “Research Process; It is essential to elaborate on how the experts were selected and which sampling method was used to select experts? It is important to clearly give detail information on methods selection for methodology section.” Our response: We appreciate the reviewer’s request for clarification. In response, we have revised the Methodology section to provide more detail on both the expert selection process and the rationale for our methodological choices. We now clarify that the expert group was selected using purposive sampling, targeting individuals with at least two years of experience in research or implementation of digital building permits (DBP) and/or digital building logbooks (DBL). Most experts were actively involved in ongoing European R&D projects related to these topics. We have added a justification for using a four-phase mixed-method approach combining literature review, expert elicitation, SDG mapping, and stakeholder validation. This approach was chosen to ensure both theoretical grounding and practical relevance. The methodological approach was designed to bridge the gap between theory and practice. It began with a structured literature review and SDG mapping, followed by expert-driven identification of relevant targets and practices. The final phase involved a hybrid workshop with 38 participants from across Europe to validate the findings. This multi-phase design ensured that the results were both evidence-based and grounded in stakeholder perspectives. Review comment: “While the work appears technically solid and well-organized, with relevant features and structured categories, the data collection and processing protocols are not described in sufficient technical detail. The article lacks the following:  ○ Clarification on whether data were normalized or standardized. ○ No mention of data cleaning, anonymization, or aggregation methods. ○ Uncertainty regarding the temporal resolution of data.  ○ The tool or platform used for data processing and visual analytics is mentioned vaguely but not named or described.” Our response: We thank the reviewer for highlighting the need for greater transparency in our data handling and processing protocols. In response, we have revised the Research process section to include the following clarifications: As the study was qualitative in nature and based on expert elicitation and workshop validation, numerical data normalisation or standardisation was not applicable. However, we have clarified this explicitly in the revised text. We now specify that no personal data was collected during the workshop. Background information was gathered anonymously using Slido, and all responses were aggregated at the group level to ensure privacy. We have clarified that the data collected reflects a single-point-in-time assessment conducted during the Sustainable Places 2024 conference on September 24, 2024. We now explicitly state that Slido was used for collecting participant background data, and Miro was used for collaborative SDG mapping during the workshop. These tools were chosen for their accessibility and ability to support hybrid participation. Review comment: “Conclusion - Were there any limitations in your research work?” Our response: Yes, we acknowledge several limitations in our research, which we have now explicitly stated in the revised Conclusions section. The study is based on expert elicitation and workshop validation, which, while rich in insight, may introduce subjectivity and limit generalisability. The workshop participants were primarily from European countries, which may limit the applicability of findings to other regions with different regulatory or technological contexts. The data reflect a single-point-in-time assessment conducted during the Sustainable Places 2024 conference. As such, the findings may not capture evolving practices or future developments in DBP and DBL. The study does not include longitudinal tracking of DBP/DBL implementation impacts, which would be valuable for assessing sustainability outcomes over time. While tools like Slido and Miro facilitated data collection and collaboration, they also constrained the depth of interaction and granularity of data that could be captured.

# Reviewer report: Reviewer response for version 1

**DOI:** 10.21956/openreseurope.20061.r52624

**Published:** 2025-04-02

**Authors:** Rupesh Kumar

**Affiliations:** 1 Jindal Global University, Sonipet, Haryana, India

Author needs to cite few important latest papers.

 Author needs to support the theory with more explanation.

 Author needs to compare the other studies to support its study with latest references.

 Author needs to check for grammatical mistakes and avoid using long sentences.

 Author needs to see their figures and tables are properly cited.

 The method used by author should be unique and give explanation as compared to other work.

 Author should check the format of using citations and references as per journal guidelines.

 Author should address all the comments given earlier and should provide organized structure of their manuscript.

Is the study design appropriate and does the work have academic merit?

Yes

Is the work clearly and accurately presented and does it cite the current literature?

Partly

If applicable, is the statistical analysis and its interpretation appropriate?

Yes

Are all the source data underlying the results available to ensure full reproducibility?

Partly

Are the conclusions drawn adequately supported by the results?

Partly

Are sufficient details of methods and analysis provided to allow replication by others?

Yes

Reviewer Expertise:

Sustainable, Analytics

I confirm that I have read this submission and believe that I have an appropriate level of expertise to confirm that it is of an acceptable scientific standard, however I have significant reservations, as outlined above.

## References

[1] : Supply chain process of olive oil industry. *International Journal of Management Practice* .2018;11(2) : 10.1504/IJMP.2018.090829 10.1504/IJMP.2018.090829

[2] : Services Redesign Strategies for Demand and Capacity Management and Impact on Company Performance. *Vision: The Journal of Business Perspective* .2022; 10.1177/09722629221107238 10.1177/09722629221107238

[3] : A digital ecosystem for sustainable fruit supply chain in Uttarakhand: a comprehensive review. *Environment, Development and Sustainability* .2023;26(5) : 10.1007/s10668-023-04142-8 13217-13252 10.1007/s10668-023-04142-8

[4] : Identification of issues in the cold chain of Indian frozen food. *International Journal of Logistics Economics and Globalisation* .2023;10(1) : 10.1504/IJLEG.2023.130236 10.1504/IJLEG.2023.130236

# Response

**Published:** 2025-07-08

**Authors:** Rita Lavikka

**Affiliations:** Built Environment and Mobility, VTT Technical Research Centre of Finland Ltd, Espoo, Uusimaa, Finland

We thank the reviewer for the constructive comments, which helped us improve our article. Below, we provide our more detailed responses to each review comment. Review comment: “Author needs to cite few important latest papers.” Our response: Thank you for suggesting that we add a few important, latest papers. We checked Scopus and Google Scholar and identified one new paper addressing the sustainability of digital building permits and logbooks, and added it to the paper: Mêda P, Fauth J, Schranz, et al.: Twinning the path of digital building permits and digital building logbooks – Diagnosis and challenges. Developments in the Built Environment. 2024. 10.1016/j.dibe.2024.100573. We also reviewed the ones suggested by the reviewer. The paper by Sharma et al. (2023) is relevant to our study, and we have added it to our paper since it examines digitalisation as an enabler of sustainability in fruit supply chains and proposes technological solutions that can contribute to sustainable horticulture practices. Please see the section Discussion. Three other papers appear to address different types of issues, not our topic: the sustainability impacts of digital building permit (DBP) processes or digital building logbooks (DBL), and strategies for achieving them. We will address each paper in more detail: The paper by Kumar & Kansara (2018) focuses on the optimisation and analysis of supply chain processes in the olive oil industry. This paper is not directly related to our paper in terms of subject matter or methodology, but they are complementary examples of how digitalisation can support sustainability in different sectors. However, as we are not exploring cross-sectoral insights on digital tools for sustainability, we will not use it as a reference, even though Kumar & Kansara’s paper is an interesting piece of research. The paper by Rajani et al. (2022) examines the relationship between selected supply chain risks, the utilisation of service redesign strategies, and their impact on companies. We couldn’t find the connection to our paper’s topic. The paper by Arora et al. (2023) examines the issues affecting the frozen food cold chain in India and proposes strategies to address these issues, without a primary focus on sustainability. Review comment: “Author needs to support the theory with more explanation.” Our response: We appreciate the reviewer's insightful comment. We elaborated on the theoretical underpinnings of how digital transformation supports sustainability in the AECO sector. Please see the Introduction section. Review comment: “Author needs to compare the other studies to support its study with latest references.” Our response: We added the reference to Sharma et al. (2023). This is the only comparable study that could be found despite conducting an extensive literature review.Otherwise, we couldn’t identify new papers. Review comment: “Author needs to check for grammatical mistakes and avoid using long sentences.” Our response: We corrected grammatical errors and condensed lengthy sentences. Review comment: “Author needs to see their figures and tables are properly cited.” Our response: We have verified that Figure 1 and Supplementary Table 1 are properly cited, and the technical editors of Open Research Europe have also confirmed this prior to the first publication. Review comment: “The method used by author should be unique and give explanation as compared to other work.” Our response: We added more explanation about the uniqueness of our method to the section Research process. Review comment: “Author should check the format of using citations and references as per journal guidelines.” Our response: Citations and references have been checked. Also, the technical editors of Open Research Europe check this before publication. Review comment: “Author should address all the comments given earlier and should provide organized structure of their manuscript.” Our response: All comments are addressed, and an organised structure of the manuscript is followed. Thank you once again for your review comments that helped us improve our paper.
